# Long-lived CD8+ T cell responses following Crimean-Congo haemorrhagic fever virus infection

**DOI:** 10.1371/journal.pntd.0006149

**Published:** 2017-12-19

**Authors:** Dominique Goedhals, Janusz T. Paweska, Felicity J. Burt

**Affiliations:** 1 Division of Virology, National Health Laboratory Service/University of the Free State, Bloemfontein, South Africa; 2 Center for Emerging Zoonotic and Parasitic Diseases, National Institute for Communicable Diseases, National Health Laboratory Service, Johannesburg, South Africa; 3 School of Pathology, Faculty of Health Sciences, University of the Witswatersrand, Johannesburg, South Africa; Center for Disease Control and Prevention, UNITED STATES

## Abstract

Crimean-Congo haemorrhagic fever virus (CCHFV) is a member of the *Orthonairovirus* genus of the *Nairoviridae* family and is associated with haemorrhagic fever in humans. Although T lymphocyte responses are known to play a role in protection from and clearance of viral infections, specific T cell epitopes have yet to be identified for CCHFV following infection. A panel of overlapping peptides covering the CCHFV nucleoprotein and the structural glycoproteins, G_N_ and G_C_, were screened by ELISpot assay to detect interferon gamma (IFN-γ) production *in vitro* by peripheral blood mononuclear cells from eleven survivors with previous laboratory confirmed CCHFV infection. Reactive peptides were located predominantly on the nucleoprotein, with only one survivor reacting to two peptides from the glycoprotein G_C_. No single epitope was immunodominant, however all but one survivor showed reactivity to at least one T cell epitope. The responses were present at high frequency and detectable several years after the acute infection despite the absence of continued antigenic stimulation. T cell depletion studies confirmed that IFN-γ production as detected using the ELISpot assay was mediated chiefly by CD8+ T cells. This is the first description of CD8+ T cell epitopic regions for CCHFV and provides confirmation of long-lived T cell responses in survivors of CCHFV infection.

## Author summary

Crimean-Congo haemorrhagic fever virus (CCHFV) is a tick-borne virus that causes haemorrhagic fever with fatalities in humans. Clinical disease has been described in Africa, Asia, Eastern Europe, the Middle East, Turkey and more recently Greece, India and Spain. In the absence of specific anti-virals and vaccines, supportive therapy is the method of treatment. Specific T cell epitopes have yet to be identified for CCHFV following infection and although antibody responses are detectable in survivors, antibody production does not always correlate with viral clearance, implying that innate and T cell immunity likely also play an important role. To further understand the role of T lymphocytes in the immune response to CCHFV infection, we aimed to determine whether memory T cell responses could be identified in survivors of CCHFV infection and to identify the viral proteins on which the T cell epitopes are located. T cell responses to CCHFV peptides were detected in this study population up to 13 years after acute CCHFV infection and provides confirmation of long-lived CD8+ T cell responses. The longevity of the responses points towards memory T cells playing an important role and implies that effective long term protection from infection may be achieved through vaccination.

## Introduction

Crimean-Congo haemorrhagic fever virus (CCHFV) is a member of the *Nairoviridae* family and has a tripartite, single-stranded, negative sense RNA genome [1–3]. The three segments are referred to as the large (L), medium (M) and small (S) segments [4]. The L segment encodes the viral RNA dependant RNA polymerase which is responsible for mRNA synthesis and replication of the RNA genome [5]. The M segment encodes a number of non-structural proteins and two structural glycoproteins, G_N_ and G_C_ [6,7]. The structural glycoproteins are responsible for attachment to host cell surface receptors and therefore determine the host range and cell tropism and are also the targets for neutralizing antibodies. The viral nucleoprotein, encoded by the S segment, binds the RNA segments for the formation of ribonucleoprotein complexes and shows endonuclease activity, although the role of this activity in CCHFV infection is not yet clear [8,9].

CCHFV is the only member of the CCHFV serogroup of medical importance. The other members of the group, Hazara virus and Khasan virus, have not been associated with disease in humans [10–12]. CCHFV infection in humans is associated with haemorrhagic fever and is fatal in up to 30% of cases [13]. The principal vectors of the virus are ticks belonging to the genus *Hyalomma* [14]. The distribution of disease follows that of the principal vector of the virus [13,15]. Clinical disease is well described in Africa, Asia, Eastern Europe and the Middle East and has recently emerged in Turkey, Greece, India and Spain [16–19]. With nearly 10 000 human cases reported to the Ministry of Health in Turkey between 2002 and December 2015, as well as expanding areas of endemicity, the development of effective preventative and therapeutic measures have now become a priority [20]. To this end, the correlates of protection against CCHFV need to be determined.

Antibody responses develop within 7–9 days of infection and include a transient IgM response, and an IgG response which likely persists for life [21,22]. In cases where an antibody response is not detectable by day nine, a fatal outcome is almost invariably seen [21]. However studies looking at viral load and antibody titre deduced that the detection of IgM had no influence on clearance of the virus or outcome, and viral load decreased independently of IgG [23]. Hence, antibody production does not always correlate with viral clearance, implying that innate and T cell immunity likely also play an important role in viral clearance [24]. In addition, neutralizing antibodies do not always confer protection *in vivo*, while non-neutralizing antibodies may confer protection through mechanisms such as antibody-dependent cell-mediated cytotoxicity [25]. The nucleoprotein induces antibody production and this region is widely used as the antigenic target in enzyme-linked immune-sorbent assays (ELISA) due to the robust nature of the antibody response to this protein following natural infection with CCHFV [26–34]. A study in mice suggested that neutralizing antibodies were predominantly targeted to the glycoprotein G_C_ [25]. Ideally, an effective vaccine will therefore likely require the induction of both B and T cell responses.

As the immune correlates of protection for CCHF are unknown, approaches to vaccine design have targeted the CCHF nucleoprotein or glycoproteins. Currently, the only vaccine that is available for human use is an inactivated vaccine used only in Bulgaria which was associated with a four-fold reduction in the number of CCHF cases following its introduction. The Bulgarian vaccine has been shown to induce both neutralising antibody responses and T cell responses to peptides across the length of the nucleoprotein, although immunogenicity required the administration of multiple doses [35, 36]. Data on immune responses to the M segment proteins and challenge studies are not available for this vaccine. A modified vaccinia virus Ankara vectored vaccine expressing the nucleoprotein induced cellular and humoral immune responses yet failed to protect mice from lethal disease [37]. Glycoprotein-based vaccines have been investigated with varying degrees of protection in mouse models [38,39]. The most promising approach thus far is a modified vaccinia virus Ankara recombinant vaccine expressing the viral glycoproteins which induced cellular and humoral responses and provided protection from lethal disease in mice [39,40].

There are currently no data available evaluating T cell responses following natural infection with CCHFV in human cases. To further understand the role of T lymphocytes in the immune response to CCHFV infection, we aimed to determine whether memory T cell responses could be identified in survivors of CCHFV infection and to identify the viral proteins on which the T cell epitopes are located. An overlapping peptide library was used to screen for T cell responses by IFN-γ ELISpot assay using peripheral blood mononuclear cells (PBMC) derived from survivors of previous CCHFV infection.

## Methods

### Ethics statement

Written informed consent was obtained from all participants and approval for the study was obtained from the Ethics Committee of the Faculty of Health Sciences, University of the Free State (ECUFS NR 152/06).

### Study participants

Eleven adult patients with a history of laboratory confirmed CCHFV infection were included in the study. Laboratory confirmation of CCHFV infection was performed at the National Institute of Communicable Diseases, Johannesburg, South Africa at the time of the acute illness by means of viral nucleic acid detection by reverse transcription polymerase chain reaction (RT-PCR), virus isolation, or detection of CCHFV specific antibodies. Sodium heparin blood samples were transported to the laboratory for processing within 4 to 6 hours after collection. Two additional participants were included in the study as negative controls and were selected as they had no history of CCHFV infection, exposure to CCHFV or risk factors for such exposure.

### Synthetic peptides

An overlapping peptide library containing 156 peptides consisting of 19-mers with a 9-mer overlap and spanning the nucleoprotein (482 amino acids) and the mature glycoproteins, G_N_ (292 amino acids) and G_C_ (648 amino acids), were synthesized (Mimotopes, Victoria, Australia) based on the deduced amino acid sequences of CCHFV isolate SPU103/87 (GenBank accession numbers DQ211647 and DQ211634).

### ELISpot assays

Fresh PBMC were isolated from whole blood using Ficoll-Hypaque density gradient centrifugation. IFN-γ ELISpot assays were performed using 96 well plates (MultiScreen-IP, Millipore) pre-coated with anti-IFN-γ antibody (clone 1-D1K; Mabtech) at 2μg/ml at 4°C overnight. After washing with sterile phosphate buffered saline (PBS), the plate was blocked with R10 (RPMI 1640 medium plus 1% penicillin-streptomycin, 1% L-glutamine and 10% foetal calf serum) for 2 hours at room temperature. Screening of the peptide library was performed by means of a matrix of 36 peptide pools containing 7 to 9 peptides each so that each peptide was included in two separate pools. Peptides were added at a final concentration of 5μg/ml for each peptide. PBMC were then seeded at 1x10^5^ to 2x10^5^ cells per well in R10. For positive controls, PBMC were stimulated with phytohaemaglutinin (Sigma-Aldrich) and 0.1μg/ml monoclonal antibody to human CD3 (clone CD3-2, Mabtech) in separate wells. Unstimulated PBMCs in R10 and wells with media and no PBMCs were included as negative controls. Plates were incubated for 16 hours at 37°C and 5% CO_2_. The plates were then washed 6 times with PBS and 2μg/ml of biotinylated anti-IFN-γ antibody (clone 7-B6-1, Mabtech) was added and incubated for 3 hours at room temperature. The plates were again washed with PBS and streptavidin-alkaline phosphatase conjugated antibody (Mabtech) was added and the plate incubated for 1 hour at room temperature in the dark. After a final wash, the alkaline phosphatase conjugate substrate kit (Bio-Rad) was used according to manufacturer’s instructions to detect IFN-γ producing cells. The number of spots were counted manually and results were expressed as the number of spot forming cells per million cells (SFC/10^6^ cells). A response was considered positive if it exceeded 50 SFC/10^6^ cells after subtraction of the background count from the negative controls, while negative controls were consistently below this cut-off. The peptides showing a positive response using the pool screening method were then tested individually using the same method and with a final peptide concentration of 5 μg/ml per well.

### T cell subset depletion studies

T cell subset depletion assays were performed to determine whether the positive responses obtained were predominantly due to CD4+ or CD8+ T cells. Based on the outcome of initial ELISpot screening, a patient that reacted strongly to two epitopes was selected for subset depletion assays in which PBMC isolated from the patient were depleted of CD8+ T cells using Dynabeads CD8 (Invitrogen) according to the manufacturer’s instructions. The ELISpot assay was then performed using depleted and undepeleted cells stimulated with each peptide for which the survivor had previously shown a positive reaction.

### Epitope conservation

To determine whether the identified T cell epitopes were conserved amongst geographically distinct CCHFV isolates, 40 complete S and M segment sequences were retrieved from the GenBank database. The sequences were aligned using Clustal X version 2.0 [41] and further edited using BioEdit version 7.2.3 (available at http://www.mbio.ncsu.edu/bioedit/bioedit.html).

## Results

Eleven survivors were included in the study, all of whom were Caucasian males residing in the Free State and North West provinces of South Africa at the time of CCHFV infection. The interval between CCHFV infection and sample collection for this study ranged from 10 months to 13 years. The details of the survivors are summarised in Table 1.

**Table 1:** Patient information. Summary of the patients with previous CCHFV infection from whom peripheral blood mononuclear cells were extracted for interferon gamma ELISpot assays.

Study number	Gender	Age (years)	Province	Likely route of exposure	Time since infection
01	Male	53	Free State	Tick bite	13 years
02	Male	75	Free State	Tick bite	13 years
03	Male	45	Free State	Tick bite	12 years
06	Male	59	Free State	Tick bite	5 years
07	Male	69	Free State	Tick bite	5 years
08	Male	42	North West	Veterinarian	5 years
09	Male	50	Free State	Tick bite	5 years
11	Male	56	Free State	Tick bite (veterinarian)	4 years
12	Male	70	Free State	Livestock and tick exposure	3 years
13	Male	67	Free State	Tick bite	3 years
15	Male	36	Free State	Livestock and tick exposure	10 months

**Fig 1:**
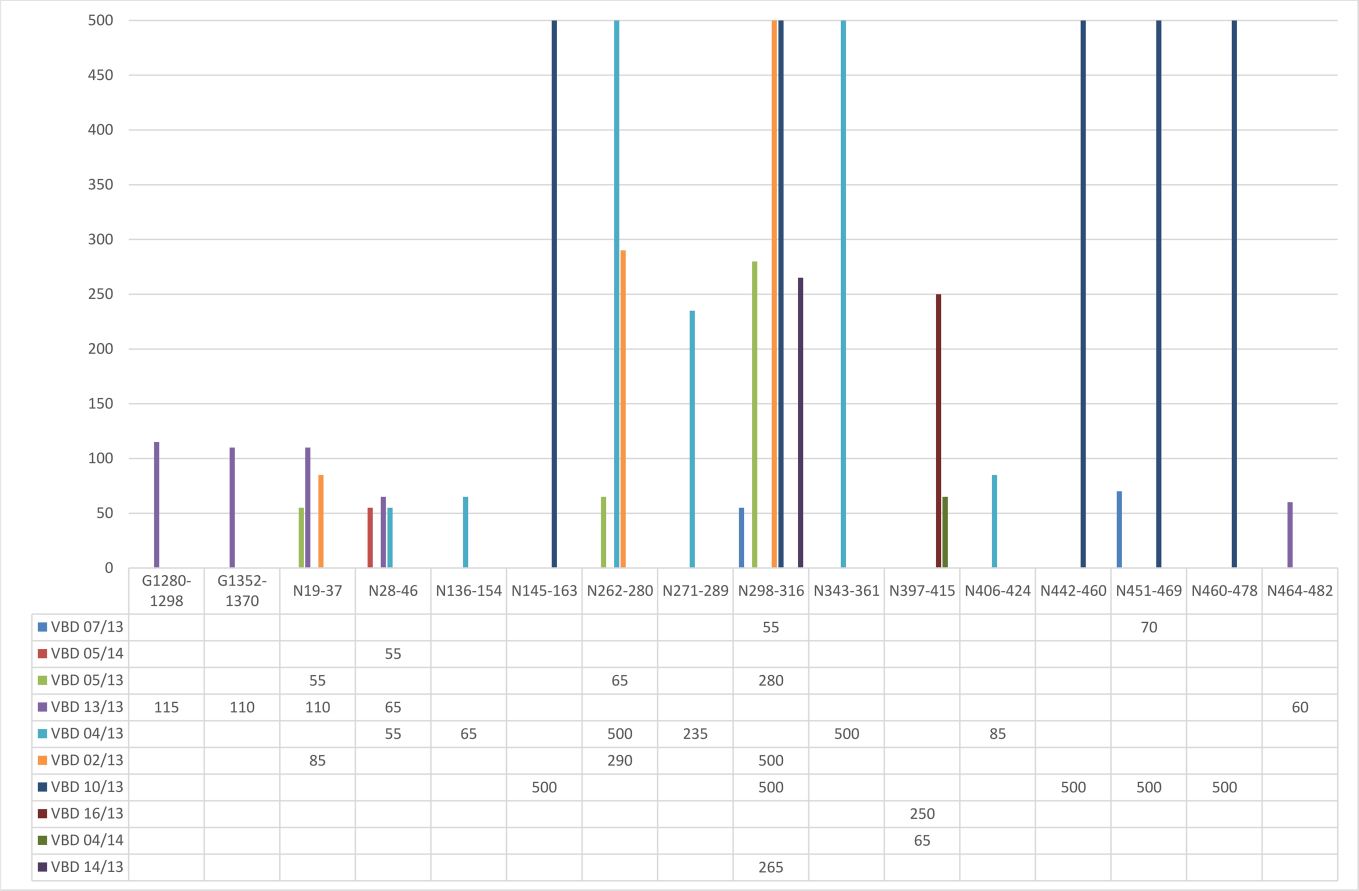
Detection of virus-specific T cell responses by IFN-γ ELISPOT assay. The magnitude of responses in spot forming cells per million (SFC/10^6^) are indicated for each of the peptides to which study participants showed a positive response.

To identify epitopic regions targeted by T cell responses, PBMCs were collected from survivors and stimulated using 36 pools containing 7–9 overlapping peptides representing the nucleoprotein and glycoproteins, G_N_ and G_C_. IFN-γ responses were detected against 16 peptides using the ELISpot assays, with all but one survivor responding to at least one peptide (Fig 1). Due to reactivity against adjacent peptides, it is likely that six of the epitopes reside within the overlapping regions, resulting in a total of ten epitopic regions identified. The details of the peptides showing positive responses are summarised in Table 2. The majority of the potential epitopes were located on the nucleoprotein, with only one patient responding to two peptides located on the glycoprotein G_C_. No region of the nucleoprotein appeared immunodominant, with the epitopes distributed throughout the length of the protein. The two glycoprotein epitopes were restricted to G_C_, starting at amino acid positions 244 and 316 respectively of the 648 amino acid long coding region of this protein. No response was detected when PBMC from two volunteers with no history of CCHF infection were tested against the 16 reactive peptides, confirming that the responses detected in the study participants likely result from previous CCHFV exposure and were not due to non-specific reactivity.

**Table 2:** Epitopic regions. Details of the peptides representing potential epitopic regions identified by ELISpot assay. Peptide names are derived from the relevant protein and the amino acid position relative to the coding regions of SPU 103/87 (DQ211647 and DQ211634). Six adjacent overlapping peptides were reactive, with each pair likely representing a single epitopic region as indicated with a superscript numeric and amino acid residues in the overlapping region are indicated in bold print.

Peptide	Amino acid sequence	Number of positive responses	Range of magnitude of responses (SFC/10^6^)
G_1280-1298_	TLHPRIEEGFFDLMHVQKV	1	115
G_1352-1370_	DGCDLDYYCNMGDWPSCTY	1	110
N_19-37_^1^	EFKKGNGLV**DTFTNSYSFC**	3	55–110
N_28-46_^1^	**DTFTNSYSFC**ESVPNLDRF	3	55–65
N_136-154_^2^	DIGFRVNAN**TAALSNKVLA**	1	65
N_145-163_^2^	**TAALSNKVLA**EYKVPGEIV	1	>500
N_262-280_^3^	DKHKDEVDR**ASADSMITNL**	3	65 - >500
N_271-289_^3^	**ASADSMITNL**LHKIAKAQE	1	235
N_298-316_	RAQGAQIDTAFSSYYWLYK	5	55 - >500
N_343-361_	KMKKALLSTPMKWGKKLYE	1	>500
N_397-415_^4^	VANPDDAAQ**GSGHTKSILN**	2	65–250
N_406-424_^4^	**GSGHTKSILN**LRTNTETNN	1	85
N_442-460_^5^	NIQDMDIVA**SEHLLHQSLV**	1	>500
N_451-469_^5^	**SEHLLHQSLV**GKQSPFQNA	2	70 - >500
N_460-478_^6^	VGKQ**SPFQNAYNVKGNATS**	1	>500
N_464-482_^6^	**SPFQNAYNVKGNATS**ANII	1	60

No clear conclusion could be reached on the effect of the time interval between CCHFV infection and sampling with regard to the magnitude and range of T cell responses. Although the two survivors with intervals of 12 and 13 years since infection respectively showed only low level responses (55–70 SFC/10^6^) to one or two peptides, the survivor with the most recent history of infection only 10 months prior to sampling also showed a low level response (65 SFC/10^6^) to a single peptide. This variation is therefore likely a consequence of individual patient responses rather than duration after infection.

**Fig 2:**
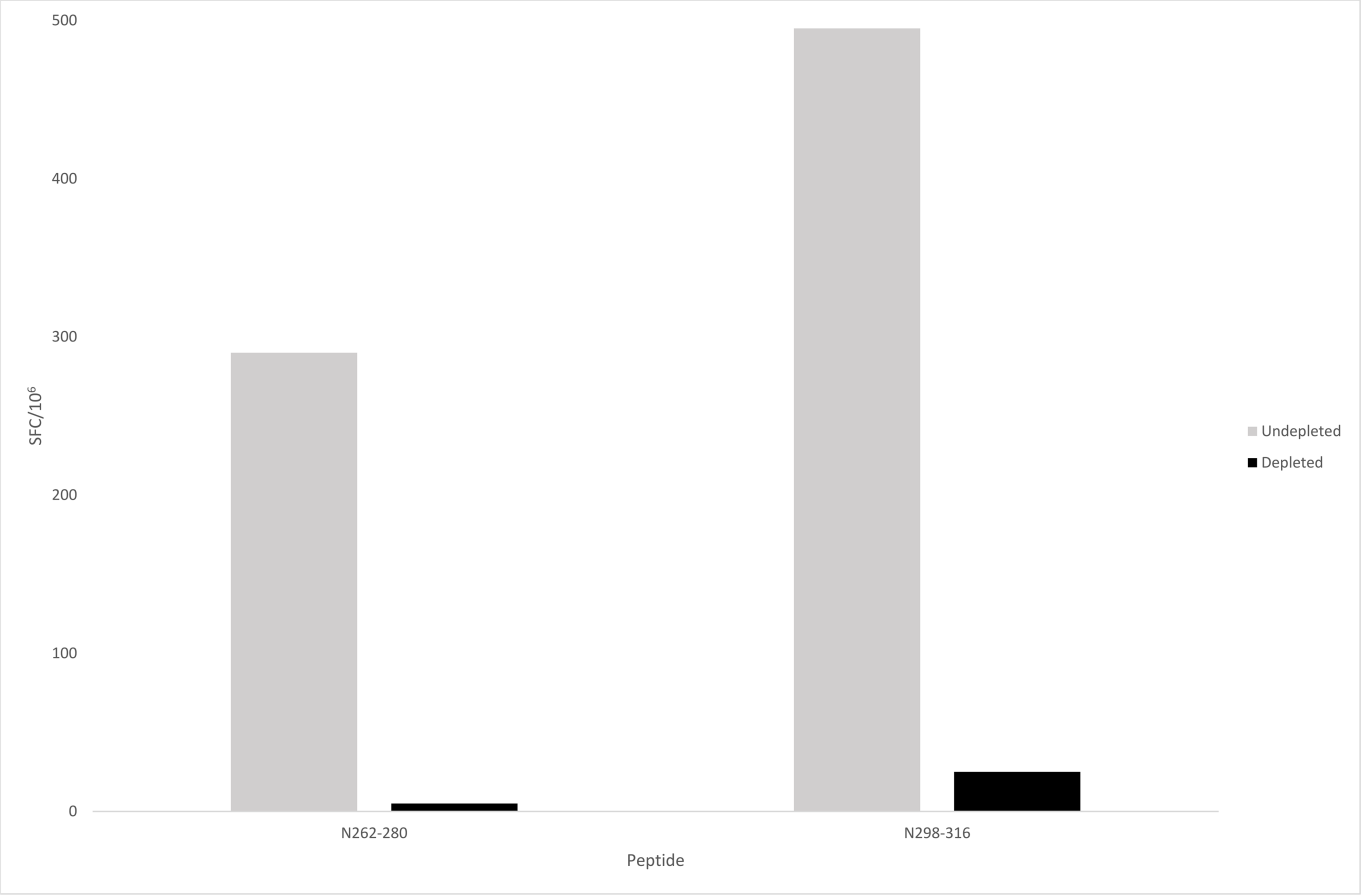
CD8+ T cell depletion assays. The magnitude of IFN-ɣ ELISpot responses in spot forming cells per million (SFC/10^6^) are depicted, before and after CD8+ T cell depletion using magnetic beads, for peptides N_262-280_ and N_298-316_.

PBMCs from three individuals had strong responses (65 - >500 SFC/10^6^) against peptide N_262-280_ and PBMCs from five individuals had significant responses (55 - >500 SFC/10^6^) against N_298-316_, hence these two peptides were selected for CD8+ depletion studies. Participant 11 was selected due to strong responses to both of these peptides, with 290 and >500 SFC/10^6^ detected respectively during the screening ELISpot testing. Following CD8+ T cell depletion, the number of SFC/10^6^ dropped to 5 and 25 respectively confirming a predominantly CD8+ T cell response (Fig 2).

Alignment of the 40 predicted amino acid sequences of the epitopic regions obtained from GenBank were relatively well conserved among geographically distinct CCHFV isolates despite representing genetically diverse groups. The global sequences showed a maximum of three amino acid differences across each 19-mer compared to SPU103/87, with the exception of isolate AP92 which showed higher variance. Amino acid sequence conservation was higher among the southern African isolates with a maximum of one amino acid difference across each 19mer. In addition to the geographical distribution, the CCHFV isolates were also grouped according to phylogenetic relatedness as described previously [42,43] and the number of amino acid differences were tabulated for each peptide (Table 3). This confirmed the higher variance of isolate AP92 (group VI), while the other groups showed a maximum of 3 amino acid differences. The amino acid alignments for each peptide are included in the supporting information files (S1 Data).

**Table 3:** Amino acid sequence conservation in epitopic regions. CCHFV isolates were grouped according to phylogenetic relatedness as described previously [42,43] and the number of amino acid differences compared to SPU 103/87 were tabulated for each reactive peptide.

	I West Africa	II DRC	III South/ West Africa	IV Asia/ Middle East	V Europe/ Turkey	VI Greece	VII Mauritania	Other
**G**_**1280-1298**_	1	1	0–1	0–1	1–2	3	1	2
**G**_**1352-1370**_	1	1	0	0–1	0–1	1	0	1
**N**_**19-37**_	0	1	0	0–1	0–1	0		
**N**_**28-46**_	1	0	0–1	0–1	2	1		
**N**_**136-154**_	1	0	0	0–2	0	2		
**N**_**145-163**_	1	0	0	0–2	0	2		
**N**_**262-280**_	2	3	0–1	0–3	2	6		
**N**_**271-289**_	2	2	0–1	0–3	0–1	4		
**N**_**298-316**_	2	2	0–1	0–1	2	2		
**N**_**343-361**_	0	0	0–1	0–1	0	0		
**N**_**397-415**_	0	0	0	0–1	0	0		
**N**_**406-424**_	1	0	0–1	0	1	1		
**N**_**442-460**_	0	0	0–1	0–1	0–1	2		
**N**_**451-469**_	0	0	0	0	0–1	0		
**N**_**460-478**_	0	0	0	0	0	0		
**N**_**464-482**_	0	0	0–1	0	0	0		

## Discussion

The present study is the first description of T cell epitopes in survivors of CCHFV infection. A library of overlapping peptides covering the nucleoprotein, G_N_ and G_C_ proteins of CCHFV was screened by ELISpot assay to determine *in vitro* induction of IFN-γ production using PBMC from survivors. The rationale for selection of these viral proteins was because they are the most likely to contribute towards a protective immune response based on knowledge from related viruses and CCHF vaccine studies [37, 44–47]. Ten probable epitopic regions residing predominantly on the nucleoprotein with only two epitopes on the glycoprotein G_C_ were identified. The predominance of nucleoprotein epitopes is similar to findings on Hantaan virus T cell epitopes [44]. The reason for this is unclear but may result from the abundance of nucleoprotein production during CCHFV replication *in vivo*. A nucleoprotein based vaccine for Rift Valley fever virus (RVF), another bunyavirus, has been shown to protect against challenge with a lethal dose of virus in a mouse model despite the absence of neutralizing antibodies [45]. This, and other RVF nucleoprotein vaccines, have been shown to induce strong memory T cell responses and an early type I interferon response which likely contribute to protection [46,47]. The CCHFV nucleoprotein represents an enticing option for vaccine design due to the greater sequence conservation of this protein and strong antibody responses, however a recombinant nucleoprotein vaccine failed to provide protection despite inducing both cellular and humoral responses [37].

No single epitope was found to be immunodominant, rather a variety of epitopes were identified in different patients. The high level of amino acid sequence conservation amongst southern African CCHFV isolates implies that this did not result from sequence variation which may result in an inability of T cells to recognise the peptides, but likely represents the HLA diversity within the patient population studied and the differing HLA restrictions of the peptides. A range of epitopes will most probably need to be included in any prospective vaccine candidates in order to induce protective T cell and antibody responses in all vaccine recipients. The current findings suggest that vaccines based on the structural glycoproteins, G_N_ and G_C_, may not induce sufficient T cell responses to provide protection in human populations. This is supported by the failure of a subunit vaccine comprised of G_N_ and G_C_ to provide protection despite inducing high levels of neutralizing antibodies [38].

Although the M segment is known to be less conserved than the S segment, with amino acid variation of up to 30.1% and 8.4% respectively amongst global isolates, differences in amino acid identity for the G_N_ and G_C_ proteins specifically are less marked at 8.5% and 4.8% respectively [42]. It is therefore unlikely that the limited responses to the glycoproteins resulted from the inability of memory cells to recognise epitopes on the synthetic peptides.

The strength of the T cell responses identified differed between peptides and between patients responding to the same peptide. The magnitude of T cell responses induced by longer peptides such as the 19mers used in this study, may be underestimated in comparison to the more effective responses induced by optimal nonamers. This increased efficiency of T cell response induction may result from direct binding of antigen presenting cells to the nonamers [44]. This phenomenon would not have influenced the outcomes of this study which aimed to identify long lived T cell responses and the location of these epitopes on viral proteins rather than to accurately quantify the magnitude of these responses.

Memory T cells, especially CD8+ cytotoxic cells, are able to rapidly proliferate and differentiate into effector cells which are an important part of long-lived protective immune responses following both vaccination and natural infection [48]. Depletion studies from one patient confirmed that the T cell responses observed following CCHFV infection in that patient resulted from CD8+ cells rather than CD4+ helper cells. Examination of additional patients and phenotype of reactive memory cells requires further confirmation.

CCHFV is transmitted to humans by tick bites, squashing of ticks with bare hands and contact with blood or tissues of infected humans and animals. Persons at high risk of exposure to CCHFV therefore include those likely to be exposed to ticks and those performing procedures resulting in exposure to blood or tissues of infected animals such as castration or slaughtering including farmers, farm workers, abattoir workers and veterinarians [49,50]. From the first recognised case of CCHFV infection in South Africa in 1981 until 2013, a total of 194 cases were recorded in this country. Of these cases, 91% occurred in males and more than 50% originated in the Free State and Northern Cape Provinces [51]. The exclusively male cohort analyzed in this study is therefore representative of the gender distribution of cases in South Africa.

T cell responses to CCHFV peptides were detected in this study population up to 13 years after acute CCHFV infection. The longevity of the responses points towards memory T cells playing an important role and support findings of the long-term presence of memory CD8+ T cells following infection with another bunyavirus, Puumala virus. This virus causes an acute infection with no known latent or chronic phase in humans, however Puumala virus specific memory T cell responses were present up to 15 years after infection despite the absence of continued antigenic stimulation or re-exposure [52]. These data imply that effective long term protection from infection may be achieved through vaccination. The MVA vectored glycoprotein vaccine which provided 100% protection in mouse challenge studies, induced both cellular and humoral responses. This vaccine expressed both the structural and non-structural glycoproteins, namely the variable mucin-like domain, GP38 and NS_M_ [39]. The T cell responses to this vaccine mapped largely to the N-terminus of G_C_ and the non-structural glycoproteins. Similarly, only two epitopes were identified on G_C_ following natural infection, with no responses to G_N_. Further studies are underway to investigate T cell responses to the non-structural domains in patients following CCHFV infection. The novel epitopic regions which were identified within the nucleoprotein of CCHFV represent the first such T cell epitopes to be described following natural infection and may play an important role in vaccine design and evaluation of vaccine immunogenicity.

## Supporting information

S1 Data Amino acid sequence alignments. Alignment of the predicted amino acid sequences of the epitopic regions obtained from GenBank for 40 geographically distinct Crimean-Congo haemorrhagic fever virus isolates.
(PDF)
