# Supplementary material for: Long-lived CD8+ T cell responses following Crimean-Congo haemorrhagic fever virus infection
Source: PLoS Negl Trop Dis. 2017 Dec 19;11(12):e0006149. doi: 10.1371/journal.pntd.0006149 (PMC5752039; doi:10.1371/journal.pntd.0006149)
Supplement: S1 Data — Alignment of the predicted amino acid sequences of the epitopic regions obtained from GenBank for 40 geographically distinct Crimean-Congo haemorrhagic fever virus isolates. (PDF) [file pntd.0006149.s001.pdf]

| | |
|---|---|
| | 10 |
| SPU103/87 | EFKKGNGLVDTFTNSYSFC |
| ArD15786 | ................... |
| ArD8194 | ................... |
| ROS/HUVLV-100 | ........M.......... |
| Kashmanov | ....E...M.......... |
| Drosdov | ....E...M.......... |
| Turkey-Kelkit06 | ........M.......... |
| Turkey200310849 | ........M.......... |
| KosovoHoti | ........M.......... |
| YL04057 | ................... |
| 79121M18 | ................... |
| C-68031 | ................... |
| NIVA118595 | ................... |
| NIVA118594 | ................... |
| NIV112143 | ................... |
| TAJ/HU8966 | ................... |
| Matin | ................... |
| Afg09-2990 | ................... |
| Oman | ................... |
| Baghdad-12 | ...........Y....... |
| SPU556/87 | ................... |
| SPU97/85 | ................... |
| SPU431/85 | ................... |
| SPU415/85 | ................... |
| SPU4/81 | ................... |
| SPU128/81/7 | ................... |
| SPU187/90 | ................... |
| SPU45/88 | ................... |
| SPU48/90 | ................... |
| SudanAl-Fulah | ................... |
| SPU383/87 | ................... |
| SPU44/08 | ................... |
| SPU130/89 | ................... |
| SPU497/88 | ................... |
| SPU18/88 | ................... |
| ArD39554 | ................... |
| SudanAB1-2009 | ................... |
| Ibar10200 | ................... |
| Congo3010 | ....S.............. |
| UG3010 | ....S.............. |
| Semunya | ....S.............. |
| AP92 | ................... |

$N_{19-37}$

| | |
|---|---|
| | 10 |
| SPU103/87 | DTFTNSYSFCESVPNLDRF |
| ArD15786 | ...........N....... |
| ArD8194 | ...........NM...... |
| ROS/HUVLV-100 | ...........N.....K. |
| Kashmanov | ...........N.....K. |
| Drosdov | ...........N.....K. |
| Turkey-Kelkit06 | ...........N.....K. |
| Turkey200310849 | ...........N.....K. |
| KosovoHoti | ...........N.....K. |
| YL04057 | ................... |
| 79121M18 | ................... |
| C-68031 | ................E.. |
| NIVA118595 | ................... |
| NIVA118594 | ................... |
| NIV112143 | ................... |
| TAJ/HU8966 | ................... |
| Matin | ................... |
| Afg09-2990 | ................... |
| Oman | ................... |
| Baghdad-12 | ..Y................ |
| SPU556/87 | ................... |
| SPU97/85 | ................... |
| SPU431/85 | .................K. |
| SPU415/85 | .................K. |
| SPU4/81 | .................K. |
| SPU128/81/7 | .................K. |
| SPU187/90 | .................K. |
| SPU45/88 | .................K. |
| SPU48/90 | .................K. |
| SudanAl-Fulah | .................K. |
| SPU383/87 | .................K. |
| SPU44/08 | .................K. |
| SPU130/89 | .................K. |
| SPU497/88 | .................K. |
| SPU18/88 | .................K. |
| ArD39554 | .................K. |
| SudanAB1-2009 | .................K. |
| Ibar10200 | ................... |
| Congo3010 | ................... |
| UG3010 | ................... |
| Semunya | ................... |
| AP92 | ...........N....... |

$N_{28-46}$

| | |
|---|---|
| | 10 |
| SPU103/87 | DIGFRVNANTAALSNKVLA |
| ArD15786 | ..................S |
| ArD8194 | ..................S |
| ROS/HUVLV-100 | ................... |
| Kashmanov | ................... |
| Drosdov | ................... |
| Turkey-Kelkit06 | ................... |
| Turkey200310849 | ................... |
| KosovoHoti | ................... |
| YL04057 | ..........T...H.... |
| 79121M18 | ..........T...H.... |
| C-68031 | ..............H.... |
| NIVA118595 | ..............H.... |
| NIVA118594 | ..............H.... |
| NIV112143 | ..............H.... |
| TAJ/HU8966 | ..............H.... |
| Matin | ................... |
| Afg09-2990 | ................... |
| Oman | ................... |
| Baghdad-12 | ................... |
| SPU556/87 | ................... |
| SPU97/85 | ................... |
| SPU431/85 | ................... |
| SPU415/85 | ................... |
| SPU4/81 | ................... |
| SPU128/81/7 | ................... |
| SPU187/90 | ................... |
| SPU45/88 | ................... |
| SPU48/90 | ................... |
| SudanAl-Fulah | ................... |
| SPU383/87 | ................... |
| SPU44/08 | ................... |
| SPU130/89 | ................... |
| SPU497/88 | ................... |
| SPU18/88 | ................... |
| ArD39554 | ................... |
| SudanAB1-2009 | ................... |
| Ibar10200 | ................... |
| Congo3010 | ................... |
| UG3010 | ................... |
| Semunya | ................... |
| AP92 | .....I....T........ |

$N_{136-154}$

| | |
|---|---|
| | 10 |
| SPU103/87 | TAALSNKVLAEYKVPGEIV |
| ArD15786 | .........S......... |
| ArD8194 | .........S......... |
| ROS/HUVLV-100 | ................... |
| Kashmanov | ................... |
| Drosdov | ................... |
| Turkey-Kelkit06 | ................... |
| Turkey200310849 | ................... |
| KosovoHoti | ................... |
| YL04057 | .T...H............. |
| 79121M18 | .T...H............. |
| C-68031 | .....H............. |
| NIVA118595 | .....H............. |
| NIVA118594 | .....H............. |
| NIV112143 | .....H............. |
| TAJ/HU8966 | .....H............. |
| Matin | ................... |
| Afg09-2990 | ................... |
| Oman | ................... |
| Baghdad-12 | ................... |
| SPU556/87 | ................... |
| SPU97/85 | ................... |
| SPU431/85 | ................... |
| SPU415/85 | ................... |
| SPU4/81 | ................... |
| SPU128/81/7 | ................... |
| SPU187/90 | ................... |
| SPU45/88 | ................... |
| SPU48/90 | ................... |
| SudanAl-Fulah | ................... |
| SPU383/87 | ................... |
| SPU44/08 | ................... |
| SPU130/89 | ................... |
| SPU497/88 | ................... |
| SPU18/88 | ................... |
| ArD39554 | ................... |
| SudanAB1-2009 | ................... |
| Ibar10200 | ................... |
| Congo3010 | ................... |
| UG3010 | ................... |
| Semunya | ................... |
| AP92 | .T................L |

$N_{145-163}$

| | $N_{262-280}$ | $N_{271-289}$ | $N_{298-316}$ | $N_{343-361}$ |
|---|---|---|---|---|
| SPU103/87 | DKHKDEVDRASADSMITNL | ASADSMITNLLKHIAKAQE | RAQGAQIDTAFSSYYWLYK | KMKKALLSTPMKWGKKLYE |
| ArD15786 | ........K......V... | ......V.......T.... | .........P...F..... | ................... |
| ArD8194 | ........K......V... | ......V.......T.... | .........P...F..... | ................... |
| ROS/HUVLV-100 | ...R....K.......... | ................... | .........P...F..... | ................... |
| Kashmanov | ...R....K.......... | ................... | .........P...F..... | ................... |
| Drosdov | ...R....K.......... | ................... | .........P...F..... | ................... |
| Turkey-Kelkit06 | ...R....K.......... | ..........P........ | .........P...F..... | ................... |
| Turkey200310849 | ...R....K.......... | ................... | .........P...F..... | ................... |
| KosovoHoti | ...R....K.......... | ................... | .........P...F..... | ................... |
| YL04057 | N.......K.......... | ................... | ................... | ..........L........ |
| 79121M18 | N.......K.......... | ................... | ................... | ..........L........ |
| C-68031 | ........K....N..... | ....N.............. | ................... | ................... |
| NIVA118595 | ........K....N..... | ....N.............. | ................... | ...........R....... |
| NIVA118594 | ........K....N..... | ....N.............. | ................... | ...........R....... |
| NIV112143 | ........K....N..... | ....N.............. | ................... | ...........R....... |
| TAJ/HU8966 | ........K....N..... | ....N.............. | ................... | ................... |
| Matin | ........K.......... | ................... | .........V......... | ................... |
| Afg09-2990 | ........K.......... | ................... | .........V......... | ................... |
| Oman | ........K.......... | ................... | .........V......... | ................... |
| Baghdad-12 | ........K....N.V... | ....N.V......V..... | .........V......... | ................... |
| SPU556/87 | ................... | ................... | ................... | ................... |
| SPU97/85 | ................... | ................... | ................... | ................... |
| SPU431/85 | ................... | ................... | ................... | ................... |
| SPU415/85 | ................... | ................... | ................... | ................... |
| SPU4/81 | ................... | ................... | ................... | ................... |
| SPU128/81/7 | ................... | ................... | ................... | ................... |
| SPU187/90 | ................... | ................... | ................... | ................... |
| SPU45/88 | ................... | ................... | ................... | ................... |
| SPU48/90 | ................... | ..........F........ | ................... | ................... |
| SudanAl-Fulah | ................... | ................... | ................... | ................... |
| SPU383/87 | ................... | ................... | ................... | ................... |
| SPU44/08 | ................... | ................... | ................... | ................... |
| SPU130/89 | ................... | ................... | ................... | ................... |
| SPU497/88 | ................... | ................... | ................... | ................... |
| SPU18/88 | ...............V... | ......V............ | ................... | ................... |
| ArD39554 | ........K.......... | ................... | ................... | .......T........... |
| SudanAB1-2009 | ................... | ................... | ................... | ................... |
| Ibar10200 | ................... | ................... | ...S............... | ................... |
| Congo3010 | ........K....G.V... | ....G.V............ | .........P...F..... | ................... |
| UG3010 | ........K....G.V... | ....G.V............ | .........P...F..... | ................... |
| Semunya | ........K....G.V... | ....G.V............ | .........P...F..... | ................... |
| AP92 | ...R....KT...N.V.S. | T...N.V.S.......... | .........P...F..... | ................... |

| | |
|---|---|
| SPU103/87 | VANPDDAAQGSGHTKSILN |
| ArD15786 | ................... |
| ArD8194 | ..D................ |
| ROS/HUVLV-100 | ................... |
| Kashmanov | ................... |
| Drosdov | ................... |
| Turkey-Kelkit06 | ................... |
| Turkey200310849 | ................... |
| KosovoHoti | ................... |
| YL04057 | ................... |
| 79121M18 | ................... |
| C-68031 | ................... |
| NIVA118595 | ................... |
| NIVA118594 | ................... |
| NIV112143 | ................... |
| TAJ/HU8966 | ..D................ |
| Matin | ........L.......... |
| Afg09-2990 | ........L.......... |
| Oman | ........L.......... |
| Baghdad-12 | ........L.......... |
| SPU556/87 | ................... |
| SPU97/85 | ................... |
| SPU431/85 | ................... |
| SPU415/85 | ................... |
| SPU4/81 | ................... |
| SPU128/81/7 | ................... |
| SPU187/90 | ................... |
| SPU45/88 | ................... |
| SPU48/90 | ................... |
| SudanAl-Fulah | ................... |
| SPU383/87 | ................... |
| SPU44/08 | ................... |
| SPU130/89 | ................... |
| SPU497/88 | ................... |
| SPU18/88 | ................... |
| ArD39554 | ................... |
| SudanAB1-2009 | ................... |
| Ibar10200 | ................... |
| Congo3010 | ................... |
| UG3010 | ................... |
| Semunya | ................... |
| AP92 | ................... |

$N_{397-415}$

| | |
|---|---|
| SPU103/87 | GSGHTKSILNLRTNTETNN |
| ArD15786 | ..............S.... |
| ArD8194 | .............SS.... |
| ROS/HUVLV-100 | .............S..... |
| Kashmanov | .............S..... |
| Drosdov | .............S..... |
| Turkey-Kelkit06 | .............S..... |
| Turkey200310849 | .............S..... |
| KosovoHoti | .............S..... |
| YL04057 | ................... |
| 79121M18 | ................... |
| C-68031 | ................... |
| NIVA118595 | ................... |
| NIVA118594 | ................... |
| NIV112143 | ................... |
| TAJ/HU8966 | ................... |
| Matin | ................... |
| Afg09-2990 | ................... |
| Oman | ................... |
| Baghdad-12 | ................... |
| SPU556/87 | ................... |
| SPU97/85 | ................... |
| SPU431/85 | ................... |
| SPU415/85 | ................... |
| SPU4/81 | ................... |
| SPU128/81/7 | ................... |
| SPU187/90 | ...........Q....... |
| SPU45/88 | ................... |
| SPU48/90 | ................... |
| SudanAl-Fulah | ................... |
| SPU383/87 | .................S. |
| SPU44/08 | ................... |
| SPU130/89 | ................... |
| SPU497/88 | ................... |
| SPU18/88 | ................... |
| ArD39554 | ................... |
| SudanAB1-2009 | ................... |
| Ibar10200 | ................... |
| Congo3010 | ................... |
| UG3010 | ................... |
| Semunya | ................... |
| AP92 | ................S.. |

$N_{404-424}$

| | |
|---|---|
| SPU103/87 | NIQDMDIVASEHLLHQSLV |
| ArD15786 | ................... |
| ArD8194 | ................... |
| ROS/HUVLV-100 | ................... |
| Kashmanov | ................... |
| Drosdov | ................... |
| Turkey-Kelkit06 | .................I. |
| Turkey200310849 | ................... |
| KosovoHoti | ................... |
| YL04057 | ................... |
| 79121M18 | ................... |
| C-68031 | ................... |
| NIVA118595 | ................... |
| NIVA118594 | ................... |
| NIV112143 | ................... |
| TAJ/HU8966 | ................... |
| Matin | ................... |
| Afg09-2990 | ................... |
| Oman | ................... |
| Baghdad-12 | ...H............... |
| SPU556/87 | ................... |
| SPU97/85 | ................... |
| SPU431/85 | ................... |
| SPU415/85 | ................... |
| SPU4/81 | ................... |
| SPU128/81/7 | ................... |
| SPU187/90 | ................... |
| SPU45/88 | ................... |
| SPU48/90 | S.................. |
| SudanAl-Fulah | S.................. |
| SPU383/87 | ................... |
| SPU44/08 | ................... |
| SPU130/89 | ................... |
| SPU497/88 | ................... |
| SPU18/88 | ................... |
| ArD39554 | ................... |
| SudanAB1-2009 | ................... |
| Ibar10200 | ................... |
| Congo3010 | ................... |
| UG3010 | ................... |
| Semunya | ................... |
| AP92 | D.K................ |

$N_{442-460}$

| | |
|---|---|
| SPU103/87 | SEHLLHQSLVGKQSPFQNA |
| ArD15786 | ................... |
| ArD8194 | ................... |
| ROS/HUVLV-100 | ................... |
| Kashmanov | ................... |
| Drosdov | ................... |
| Turkey-Kelkit06 | ........I.......... |
| Turkey200310849 | ................... |
| KosovoHoti | ................... |
| YL04057 | ................... |
| 79121M18 | ................... |
| C-68031 | ................... |
| NIVA118595 | ................... |
| NIVA118594 | ................... |
| NIV112143 | ................... |
| TAJ/HU8966 | ................... |
| Matin | ................... |
| Afg09-2990 | ................... |
| Oman | ................... |
| Baghdad-12 | ................... |
| SPU556/87 | ................... |
| SPU97/85 | ................... |
| SPU431/85 | ................... |
| SPU415/85 | ................... |
| SPU4/81 | ................... |
| SPU128/81/7 | ................... |
| SPU187/90 | ................... |
| SPU45/88 | ................... |
| SPU48/90 | ................... |
| SudanAl-Fulah | ................... |
| SPU383/87 | ................... |
| SPU44/08 | ................... |
| SPU130/89 | ................... |
| SPU497/88 | ................... |
| SPU18/88 | ................... |
| ArD39554 | ................... |
| SudanAB1-2009 | ................... |
| Ibar10200 | ................... |
| Congo3010 | ................... |
| UG3010 | ................... |
| Semunya | ................... |
| AP92 | ................... |

$N_{451-469}$

```
                            10
                   ....|....|....|....
SPU103/87          VGKQSPFQNAYNVKGNATS
ArD15786           ...................
ArD8194            ...................
ROS/HUVLV-100      ...................
Kashmanov          ...................
Drosdov            ...................
Turkey-Kelkit06    ...................
Turkey200310849    ...................
KosovoHoti         ...................
YL04057            ...................
79121M18           ...................
C-68031            ...................
NIVA118595         ...................
NIVA118594         ...................
NIV112143          ...................
TAJ/HU8966         ...................
Matin              ...................
Afg09-2990         ...................
Oman               ...................
Baghdad-12         ...................
SPU556/87          ...................
SPU97/85           ...................
SPU431/85          ...................
SPU415/85          ...................
SPU4/81            ...................
SPU128/81/7        ...................
SPU187/90          ...................
SPU45/88           ...................
SPU48/90           ...................
SudanAl-Fulah      ...................
SPU383/87          ...................
SPU44/08           ...................
SPU130/89          ...................
SPU497/88          ...................
SPU18/88           ...................
ArD39554           ...................
SudanAB1-2009      ...................
Ibar10200          ...................
Congo3010          ...................
UG3010             ...................
Semunya            ...................
AP92               ...................
```

$N_{460-478}$

```
                            10
                   ....|....|....|....
SPU103/87          SPFQNAYNVKGNATSANII
ArD15786           ...................
ArD8194            ...................
ROS/HUVLV-100      ...................
Kashmanov          ...................
Drosdov            ...................
Turkey-Kelkit06    ...................
Turkey200310849    ...................
KosovoHoti         ...................
YL04057            ...................
79121M18           ...................
C-68031            ...................
NIVA118595         ...................
NIVA118594         ...................
NIV112143          ...................
TAJ/HU8966         ...................
Matin              ...................
Afg09-2990         ...................
Oman               ...................
Baghdad-12         ...................
SPU556/87          ...................
SPU97/85           ...................
SPU431/85          ...............V...
SPU415/85          ...............V...
SPU4/81            ...................
SPU128/81/7        ...................
SPU187/90          ...................
SPU45/88           ...................
SPU48/90           ...................
SudanAl-Fulah      ...................
SPU383/87          ..................V
SPU44/08           ..................V
SPU130/89          ..................V
SPU497/88          ...................
SPU18/88           ...................
ArD39554           ...................
SudanAB1-2009      ...................
Ibar10200          ...................
Congo3010          ...................
UG3010             ...................
Semunya            ...................
AP92               ...................
```

$N_{464-482}$

```
                                  10
                         ....|....|....|....
SPU103/87                TLHPRIEEGFFDLMHVQKV
ArD15786                 ....K..............
ArD8194                  ....K..............
ROS-HUVLV-100            ....KV.............
Kashmanov                ....KV.............
Drosdov                  ....K..............
Turkey-Kelkit06          ....K..............
Turkey200310849          ....K..............
KosovaHoti               ....K..............
YL04057                  ....K.............I
79121M18                 ....K.............I
C-68031                  ................K..
NIVA118595               ...................
NIVA118594               ...................
NIV112143                ...................
TADJ-HU8966              ...................
Matin                    ...................
Afg09-2990               ....K..............
Oman                     ...................
Baghdad-12               ...................
SPU556/87                ...................
SPU97/85                 ...................
SPU431/85                .................R.
SPU415/85                .................R.
SPU4/81                  ...................
SPU128/81/7              ...................
SPU187/90                ...................
SPU45/88                 .................N.
SPU48/90                 ...................
SudanAl-Fulah3-2008      ...................
SPU383/87                ...............I...
SPU44/08                 ...............I...
SPU130/89                ...............I...
SPU497/88                ...............I...
SPU18/88                 ...............I...
ArD39554                 .....V.............
SudanAB1-2009            ...................
IbAr10200                ...................
Congo3010                ....K..............
UG3010                   ....K..............
Semunya                  ....K..............
AP92                     ....K..D.Y.........
```

$G_{1280-1298}$

```
                                  10
                         ....|....|....|....
SPU103/87                DGCDLDYYCNMGDWPSCTY
ArD15786                 .......F...........
ArD8194                  .......F...........
ROS-HUVLV-100            ...................
Kashmanov                ...................
Drosdov                  ............N......
Turkey-Kelkit06          ...................
Turkey200310849          ...................
KosovaHoti               ...................
YL04057                  .......F...........
79121M18                 .......F...........
C-68031                  .................S.
NIVA118595               ...................
NIVA118594               ...................
NIV112143                ...................
TADJ-HU8966              ...................
Matin                    .................S.
Afg09-2990               ...................
Oman                     ...................
Baghdad-12               ...................
SPU556/87                ...................
SPU97/85                 ...................
SPU431/85                ...................
SPU415/85                ...................
SPU4/81                  ...................
SPU128/81/7              ...................
SPU187/90                ...................
SPU45/88                 .................S.
SPU48/90                 ...................
SudanAl-Fulah3-2008      ...................
SPU383/87                .................S.
SPU44/08                 .................S.
SPU130/89                .................S.
SPU497/88                .................S.
SPU18/88                 .................S.
ArD39554                 ...................
SudanAB1-2009            ...................
IbAr10200                ...................
Congo3010                .......F...........
UG3010                   .......F...........
Semunya                  .......F...........
AP92                     .......F...........
```

$G_{1352-1370}$
